# Nutraceutical Preventative and Therapeutic Potential in Neuroblastoma: From Pregnancy to Early Childhood

**DOI:** 10.3390/life12111762

**Published:** 2022-11-02

**Authors:** Maddalena Sbaffone, Marianna Ruggieri, Michela Sebastiano, Andrew Reay Mackay, Veronica Zelli, Antonietta Rosella Farina, Lucia Annamaria Cappabianca

**Affiliations:** Department of Biotechnological and Applied Clinical Sciences, University of L’Aquila, 67100 L’Aquila, Italy

**Keywords:** nutraceuticals, neuroblastoma, pregnancy, therapy, prevention

## Abstract

Neuroblastoma (NB) is a highly malignant embryonic extracranial solid tumor that arises from sympathoadrenal neuroblasts of neural crest origin. In addition to genetic factors, NB has been linked to maternal exposure to a variety of substances during pregnancy. Recent interest in the potential of nutrients to prevent cancer and reduce malignancy has resulted in the identification of several nutraceuticals including resveratrol, curcumin, and molecular components of garlic, which together with certain vitamins may help to prevent NB development. As NBs arise during fetal development and progress during early childhood, specific NB inhibiting nutraceuticals and vitamins could enhance the preventative influence of maternal nutrition and breast feeding on the development and early progression of NB. In this article, we review NB inhibitory nutraceuticals and vitamins, their mechanisms of action and expound their potential as maternal nutritional supplements to reduce NB development and progression during fetal growth and early childhood, whilst at the same time enhancing maternal, fetal, and infant health.

## 1. Introduction

Neuroblastoma (NB) is the most common pediatric extracranial solid tumor and accounts for approximately 8% of all pediatric cancers. NBs are diagnosed in approximately 1400 children per year in Europe, 140 children per year in Italy, and 800 children per year in the USA [1,2,3] and are responsible for approximately 15% of all pediatric cancer-related deaths [2]. These highly aggressive heterogeneous tumors are more frequently diagnosed in advanced stage and despite aggressive multimodal therapeutic approaches, they exhibit frequent post-therapeutic relapse associated with low survival rates, making an effective cure for advanced stage NB highly challenging [4].

NBs arise from immature sympathoadrenal cells of neural crest origin [5,6] and exhibit a high degree of heterogeneity and variable clinical courses ranging from spontaneous regression to therapy-resistant metastatic disease. Overall survival rates range from 85–90% in low- and intermediate-risk NBs to less than 50% in high-risk NBs [5]. Current multimodal NB therapy consists of surgery, chemotherapy, radiotherapy, and autologous stem cell transplant, which are associated with significant side effects including reversible myelosuppression, kidney and cardiotoxicity, genetic damage, and hearing impairment [7]. Considering the difficulty in treating advanced stage NBs, recent interest has also focused on the cancer therapeutic and preventative properties of natural dietary compounds. This has resulted in the identification of several nutraceuticals and vitamins with potent tumor inhibitory activity [8], suggesting that dietary supplementation with anticancer nutraceuticals and vitamins may not only improve cancer prevention, but also hamper progression. In this article, we review nutraceuticals and vitamins that exhibit NB inhibitory activity, their mechanisms of action (pro-apoptosis, proliferation inhibiting, pro-differentiation, and chemotherapy efficacy promoting) and their potential use as maternal dietary supplements during pregnancy and breast-feeding in preventing the development, enhancing spontaneous regression, and reducing early progression of fetal and neonatal NBs during pregnancy and breast feeding. 

## 2. Neuroblastoma

Pediatric NBs represent 8–10% of childhood cancers and account for approximately 15% of cancer-related childhood deaths [9]. NBs are slightly more frequent in boys, but no significant differences have been reported in the incidence of NB between ethnicities [10]. The incidence of NB in children less than 1 year old is estimated at 65 cases per million and in children under 15, 11–13 cases per million [11]. Approximately 30% of NBs are diagnosed within the first year of life and the remainder diagnosed before the age of 5 [10]. NBs in adolescents and young adults are rare but carry poor prognosis [12]. NB patients of less than 18 months exhibit more frequent spontaneous regression and enhanced survival rates (86.0% 3-year overall survival and 84.6% 5-year overall survival), whereas survival rates are lower in older individuals (45.9% 3-year overall survival and 36.3% 5-year overall survival) [11,12].

### 2.1. NB Risk Factors and Pathogenesis

Despite extensive experimental studies, the etiology of NB remains to be elucidated. Hereditary familial NBs are rare (1–2% of all cases) and have been attributed to germline gain of function mutations in anaplastic lymphoma kinase (ALK) [13,14] and germline loss of paired like homeobox 2B (PHOX2B) [13]. NBs develop during fetal development, supporting the hypothesis that maternal exposure to certain substances and conditions prior to or during pregnancy may be important in NB development and early progression. Within this context, the exposure of pregnant women to permanent hair dye during the first and second trimesters is associated with a higher risk of NB development; maternal workplace exposure to solvents [15] and air stable polycyclic aromatic hydrocarbons has also been linked to increased risk of NB development [16]. Furthermore, smoking, alcohol, or the use of drugs during pregnancy have been implicated in increasing the risk of fetal NB development, and nicotine has been shown to promote NB cell proliferation in vitro by inducing the expression of brain derived neurotrophic factor and its receptor, Tropomyosin receptor kinase B (TrkB). However, further studies are required to confirm the potential causal relationship during pregnancy, the doses required to enhance risk, and whether the co-consumption of nicotine, alcohol, and illicit drugs increase the risk of NB development [17,18].

On the other hand, breastfeeding from 3 to 13 months has been shown to reduce NB development by providing nerve and insulin-like growth factors that inhibit sympathoadrenal neuroblast proliferation and promote their differentiation during the neonatal period [19]. 

NBs arise from sympathoadrenal progenitor cells of neural crest origin that normally give rise to the sympathetic ganglia, sympathetic neurons, and adrenal chromaffin cells [5,6,20,21]. NB cells do not respond to differentiation signals and are blocked in a de-differentiated, stress-resistant proliferating state [22]. This state is facilitated by activated oncogenes including MYCN, which is amplified in approximately 20% of primary NBs and carries poor prognosis [23,24]; the hypoxia-regulated oncogenic alternative TrkAIII splice variant of the neurotrophin receptor tropomyosin-related kinase A (TrkA), which exhibits spontaneous intracellular activation and is expressed in advanced stage, relapsed, and metastatic NBs [25,26], aberrant neurotrophin receptor TrkB expression [27], and oncogenic ALK activation [28], to name a few.

### 2.2. Diagnosis, Risk-Classification, and Current Therapies

The median age of NB diagnoses is 18 months, with 90% of NBs diagnosed prior to the age of 5. Patients <18 months of age exhibit more frequent spontaneous NB regression, whereas NBs in older children are more aggressive and associated with lower survival rates [2,13]. Primary NBs may occur throughout the sympathetic nervous system but develop more frequently in the adrenal glands [21]. NB diagnosis combines the histological examination of biopsies with imaging and biochemical profiling, and at the histological level, NBs appear as small, round, undifferentiated intensely stained cells [29].

NBs exhibit highly heterogeneous biological and clinical characteristics [30]. NB risk-classification, established by the International Neuroblastoma Risk Group (INRG), is based on precise prognostic factors (INRG stage, age at diagnosis, histologic category, grade of tumor differentiation, MYCN alterations, 11q aberrations and ploidy) and delineates pretreatment risk-groups [31]. Low- and intermediate-risk NB patients have the best prognosis and, in most cases, surgery alone is curative. Therapeutic strategies for high-risk NBs are divided into three treatment blocks: induction, consolidation, and post-consolidation therapy. The induction phase involves the use of chemotherapeutic agents (cisplatin, cyclophosphamide, doxorubicin, etoposide, topotecan, vincristine) and resection of the primary tumor. Consolidation therapy consists of high-dose chemotherapy in combination with external-beam radiotherapy, and autologous stem cell transplant [32], and post-consolidation therapy with anti-ganglioside 2 immunotherapy and cis-retinoic acid is used to treat minimal residual disease [31]. Conventional therapeutic approaches for high-risk patients are rarely curative and are associated with frequent relapses and low overall survival rates. Recent advances in the molecular mechanisms involved in NB pathogenesis and progression has resulted in the development of novel targeted therapies against activated ALK and Trk oncogenes, with the aim of improving the quality of life and overall survival of patients with advanced stage refractory and metastatic NB, and are currently in clinical trials [33]. A recent article has reported an exceptional durable response (of >5 years) to compassionate targeted entrectinib ALK/TrkA inhibitory therapy in an infant with relapsed refractory metastatic NB within a context of exhausted therapeutic options [34]. Considering that an effective cure for advanced stage NB is highly challenging, recent research efforts have also begun to focus on the potential of maternal nutraceutical and vitamin supplements to prevent NB development and impair early NB progression.

## 3. Nutraceuticals and Their Role in Neuroblastoma

The term “nutraceutical” was coined in 1989 by Stephen De Felice, who defined a nutraceutical as a “food, or parts of a food, that provides medical or health benefits, including the prevention and/or treatment of a disease”. Nowadays, nutraceuticals are considered to be natural molecular dietary alternatives to pharmaceuticals that provide physiological benefits [35]. Several nutraceuticals have been shown to exhibit anticancer properties including the inhibition of tumor cell invasion and metastatic progression [36], without detrimentally affecting normal cells [8]. Due to their biological anti-cancer activities, interest is increasing in the potential use of nutraceutical supplements in association with conventional treatments for both cancer prevention and anti-cancer therapy. Nutraceuticals exhibiting inhibitory activity in NB models in vitro and in vivo are listed in Table 1.

The absorption of nutrients required for fetal growth, development, and health originate from maternal nutrition and arrive at the fetus via the placenta. Both mother and fetus, therefore, are in potential danger of experiencing micronutrition deficiency during the period of gestation due to fetal growth requirements. For this reason, maternal nutrient supplementation is frequently prescribed in order to offset these potentially adverse outcomes for both mother and child, should micronutrient levels fall below a critical level. These supplements contain all of the most important components for the healthy development of the fetus [63]. 

**Figure 1:**
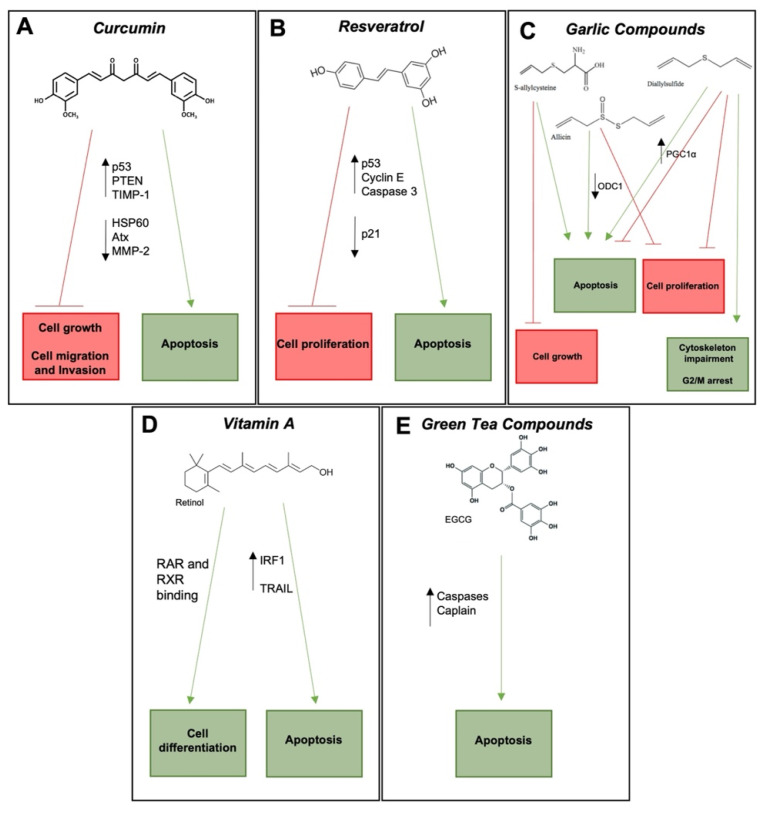
Schematic representation of the main molecular mechanisms responsible for the nutraceuticals’ anti-cancer activity in NB.

In the following sections, we review relevant nutraceuticals with NB inhibitory activity, with particular reference to curcumin, resveratrol, garlic compounds, vitamin A, green tea, and other compounds such as caffeine, berberine, and bergamot. We review the main molecular mechanisms through which these nutraceutical compounds exert their NB inhibitory activity (Figure 1). We also review the importance of maternal multivitamin and multiple-micronutrient supplements to offset nutritional deficiencies, with the aim of not only providing dietary information for optimizing maternal health and fetal development during gestation and breast feeding, but also to lower the potential risk of developing NB.

### 3.1. Curcumin

The natural polyphenol curcumin (diferuloylmethane) is the most important nutraceutical component extracted from the rhizome of native Indian plant turmeric (*Curcuma longa*) [64], commonly used for its characteristic color and flavor in curries and mustards [65,66]. Tumeric preparations contain carbohydrates, proteins, essential oils, fat, minerals, curcuminoids, and traces of vitamins [65]. The curcuminoids include curcumin, dimethoxycurumin, and bis-dimethoxycurcumin, of which curcumin is the most abundant [64]. Curcumin exists in tautomeric bis-keto and enolate forms, and at acid and neutral pH, is mainly in a hydrogen donating bis-keto form while at basic pH, it is mainly in enolic form [67]. The therapeutic potential of curcumin stems from reports of anticancer [68], anti-inflammatory [69,70], antioxidant [71], and antimicrobial activity [72]. Curcumin’s anti-cancer properties include the inhibition of tumor promotion, tumor-associated angiogenesis, and tumor growth [73]. However, curcumin exhibits poor bioavailability due to low absorption and rapid metabolism, and is rapidly eliminated from tissues and blood, regardless of the administration route [74]. Co-administration with adjuvants and nano-formulations have been developed to overcome these problems [75]. 

In NB models, curcumin inhibits MYCN-amplified NB cell proliferation and induces P53-dependent apoptosis (Figure 1A) [37]. Curcumin also promotes NB cell apoptosis by upregulating phosphatase and tensin homolog (PTEN) expression, decreasing phosphorylated Akt levels and increasing Foxo3a nuclear translocation, resulting in pro-apoptotic p27, Bim, and Fas-L expression [38]. In addition, curcumin reduces HSP60 expression in NB cells, which is involved in pathogenesis and progression and reduces HSP60 S-nitrosylation, increasing the folding capacity, and implicating HSP60 in curcumin anti-cancer activity [39]. Curcumin also inhibits NB cell migration [8], motility factor autotaxin (Atx) expression in MYCN-amplified and non-amplified NB cells [40], and matrix metalloproteinase-2 (MMP-2) expression and activates the MMP inhibitor TIMP-1 [41], which together characterize curcumin as a potent inhibitor of NB cell migration and invasion.

### 3.2. Resveratrol

The polyphenolic stilbene phytoalexin resveratrol (3,5,4′-trihydroxystilbene) is produced in damaged plants and is abundant in grapes, wine, peanuts, tea, and some berries [76,77]. Red grapes are a principal source of resveratrol, and resveratrol concentrations in red wine range from 1.5 to 3 mg L-1 [78]. Resveratrol exists in cis and trans forms, and trans-resveratrol is the more stable and abundant isoform [77]. The majority of studies have focused on the biological properties of trans-resveratrol [79]. The beneficial properties of dietary resveratrol include antioxidant, cardio-protective, and chemo-protective activity. In addition, resveratrol exhibits anti-inflammatory, antiviral, neuroprotective [80], and anti-cancer activity. Resveratrol exhibits tumorigenesis inhibitory activity in vitro and in vivo that is consistent with a cancer preventing function [81,82], and could be used in combination with standard chemotherapy because of its antioxidant and anti-inflammatory properties [82].

In NB models, resveratrol is cytotoxic to NB cells and induces NB cell apoptosis associated with caspase 3 activation (Figure 1B). Resveratrol promotes cell cycle arrest in S phase, probably by downregulating p21 and upregulating cyclin E expression, and arrests NB growth and increases survival in a mouse NB model [42]. Resveratrol also promotes mitochondrial permeability, resulting in mitochondrial cytochrome c and Smac/DIABLO release, resulting in apoptosis through the intrinsic pathway [43]. Resveratrol also inhibits proliferation and induces apoptosis in NB B65 cells through a SIRT1 independent mechanism, consistent with p53 activation and the inhibition of oncogenic signal transduction [44]. The antioxidant activity of resveratrol prevents ROS production and suppresses intra-mitochondrial activation of the oncogenic alternative TrkAIII splice variant in NB cells under conditions of ER stress, reducing stress-resistance, cell survival, and protective glycolytic metabolic adaptation [45].

The combined activity of resveratrol and immune-cytokines has also been investigated in a mouse NB model. Treatment with immune-cytokines alone arrested NB progression, which progressed after the cessation of treatment. Treatment with resveratrol alone resulted in primary tumor regression, but relapse with metastatic progression following the cessation of treatment. In contrast, treatment with resveratrol and immuno-cytokines resulted in primary tumor regression, long-term survival in 61% of mice, and the absence of metastatic progression [83]. This illustrates the need to further evaluate the NB inhibitory effects of resveratrol in combination with other drugs.

### 3.3. Garlic Compounds

Garlic (*Allium sativum* L. Fam. *Liliacee*) is a native plant of middle Asia, the beneficial effects of which have been known for over 5000 years. In ancient times, garlic was used by many populations, such as the Chinese, Israelis, Greeks, Egyptians to cure hemorrhoids, rheumatism, cough, skin disease, fever, and other diseases [84,85]. Today, garlic is known for its beneficial properties as an antioxidant [86], antibacterial agent [87], and for its anti-hypertensive [88] and anti-cancer properties [89]. Of the more than 2000 bioactive molecules present in garlic, the medicinal properties of garlic have largely been attributed to molecular organosulfur compounds [90,91], of which alliin is the most interesting. 

In chopped or crushed garlic, the non-proteinogenic amino acid alliin is converted into allicin by the release of alliinase, and is then rapidly transformed into ajoene, diallyl sulfide (DAS), and diallyl disulfide (DADS) [84]. Garlic also contains smaller amounts of biologically active g-glutamyl-S-allylcysteine (GSAC), S-methylcysteine sulfoxide (methiin), S- trans-1-propenylcysteine sulfoxide, and S-2-carboxypro-pylglutathione and S-allylcysteine (SAC) [92].

However, the antitumoral effects of many molecular garlic components have not been studied. Allicin induces apoptosis and inhibits proliferation in NB cells by downregulating ornithine decarboxylase (ODC1), a direct transcriptional target of the metastasis promoting oncogenes c-MYC and MYCN (Figure 1C) [46]. When combined with cyclophosphamide, allicin exhibits greater anti-tumor activity than cyclophosphamide alone in a mouse NB model [92]. The antitumoral effects of the garlic component SAC has also been evaluated in NB cells. SAC promotes apoptosis by inducing mitochondrial permeability and inhibits cell growth [47]. The allicin metabolite DADS induces mitochondrial apoptosis and inhibits proliferation in SH-SY5Y NB cells [48], and the pro-oxidant ROS producing activity of DADS also leads to cytoskeletal impairment, cell cycle arrest in G2/M, apoptosis, protein phosphatase 1 (PP1) activation, and subsequent Tau dephosphorylation [49]. DADS-induced ROS production, however, activates peroxisome proliferator-activated receptor-gamma co-activator 1 alpha (PGC1α), which exhibits both cancer promoting and anti-cancer activity, and in NB cells induces mitochondrial biogenesis with anti-apoptotic effects, consistent with NB promoting potential [50]. The activity of DADS in NB, therefore, should be better understood.

### 3.4. Vitamin A and Retinoids

Vitamin A is an essential liposoluble micronutrient, the precursors of which are the provitamin A forms β-carotene e β-canthaxanthin. Forms of Vitamin A include all-trans-retinol, retinal, and retinoic acid (RA), collectively known as retinoids [93,94]. Animal products such as liver, oil, milk, and eggs are the main dietary sources of vitamin A (as vitamin A esters combine with long-chain fatty acids) [95,96], whereas vegetables and fruits such as carrots, tomatoes, spinach, lettuce, parsley, chilies, apricots, and mango, are sources of provitamin A carotenoids [96]. Humans do not synthesize retinoids and are, therefore, dependent upon dietary vitamin A [97]. Following intestinal absorption, retinol is transported by chylomicrons to the liver, which is the primary retinol (retinyl esters) storage site. Retinol is subsequently exported via retinol-binding proteins (RBPs) via the bloodstream to target tissues [93,95]. 

Vitamin A is essential for physiological processes such as sight, cutaneous integrity, immune system function, reproduction, and fetal development (organogenesis) [98] and has pleiotropic functions due to its biologically active isoforms. Vitamin A deficiency or excess is associated with characteristic symptoms and diseases., including night-blindness and corneal ulcers. Excess vitamin A causes osteosclerosis and teratogenic effects including organ malformation and altered organogenesis [99]. Vitamin A concentration, therefore, must be maintained within a strict range [100]. Vitamin A supplementation is commonly combined in multiple micronutrient integration and rarely alone [63]. 

Retinoids, as cancer preventing and anti-cancer agents, have been evaluated in NB as apoptosis inducers and pro-differentiation agents (Figure 1D) [94]. All-trans retinoic acid differentiates NB cells in vitro [51] and promotes neuritogenesis consistent with NB cell neural differentiation [101,102]. RA binds nuclear retinoic acid receptors (RARs) and retinoid X receptor (RXR), resulting in the transcription of retinoic acid responsive target genes [99]. Furthermore, retinoids also activate the transcription factor IRF1, a tumor suppressor involved in activating the tumor-selective death ligand TRAIL, leading to apoptosis through the extrinsic apoptotic pathway [103].

Retinoids (particularly 13-cis-RA) are used in high-risk NB as apoptosis and differentiation inducing agents in maintenance therapy for minimal residual disease [104]. Furthermore, the stereoisomer 9-cis-RA has been shown to induce greater differentiation in NB amplified NMYC cells [53]. However, despite its anti-NB activity in a human NB rat xenograft model, 9-cis-RA exhibits higher cytotoxicity, lower bioavailability, and a short half-life, limiting clinical use [52]. The synthetic retinoid fenretinidine is currently in clinical trials [101,104]. Vitamin A is, therefore, considered as a powerful potential NB preventive agent when taken during pregnancy, as discussed below.

### 3.5. Green Tea Polyphenols

Green tea is a preparation obtained from the dried leaves of the plant *Camellia sinensis*, which contains high levels of the ubiquitous plant polyphenol catechins and expounds numerous health benefits [105,106]. Green tea polyphenols include epicatechin, epigallocatechin (EGC), epicatechin-3-gallate, and epigallocatechin-3-gallate (EGCG) [106], of which EGCG is the most abundant anti-cancer component [55] (Figure 1E). EGCG has been characterized as a potential growth inhibitor in several types of cancer including NB [105], and in SH-SY5Y NB cells induces apoptosis through caspase and calpain activation [54]. In a BE (2)-C NB cell tumor sphere model of high-risk NB, EGCG prevents the formation of tumor-initiating cells, which are considered to be responsible for tumor relapse and therapeutic resistance [55]. EGCG, furthermore, has been shown to improve the anti-tumor efficacy of the rexinoid, 6-OH-11-O-hydroxyphenanthrene [56]. EGCG, however, regulates many signaling pathways and, therefore requires further investigation in order to fully understand the mechanisms through which it exerts its anti-cancer activity [55].

### 3.6. Other Compounds

With respect to other nutraceutical molecules with potential anti-NB activity (see Table 1), berberine (BBR) is a natural alkaloid present in the rhizomes and roots of several plants including *Captis chinensis*, *Hydrastis canadensis*, *Berberis aquifolium*, *Berberis aristata*, *and Berberis vulgaris*. In NB in vitro and in vivo models, treatment with BBR leads to a reduction in tumor growth and regulates both differentiation and stemness [62,107]. In addition, BBR induces p53-dependent apoptosis in human SK-N-SH and SK-N-MC NB cell lines [60]. The in vitro cytotoxicity of BBR in cancer cells has also been reported to be enhanced when combined with arsenic trioxide (As_2_O_3_), a chemotherapeutic drug approved for the treatment of relapsed NB. BBR combined with As_2_O_3_ induces the apoptosis of SH-SY5Y NB cells by increasing the production of ROS and promoting the fragmentation of DNA, resulting in caspase activation and cell death [61]. 

The natural alkaloid caffeine, present in coffee, cocoa, tea, cola, guarana, and mate plants, is another potential NB inhibitory nutraceutical. At high concentrations, caffeine has been shown to induce apoptosis in SK-N-MC NB cells via a capase-3 dependent mechanism [59]. 

Bergamot, contained within the peel of *Citrus bergamia*, a fruiting plant of the Rutaceae family native to the Southern coast of Calabria (Italy), also exhibits NB inhibitory potential. The essential oil purified from bergamot (BEO) has been shown to reduce the in vitro tumorigenicity of SH-SY5Y NB cells, linked to p53 phosphorylation, enhance the expression and activation of the pro-apoptotic factor BAX, reduce the expression of the anti-apoptotic factor Bcl-2, and reduce the phosphorylation of p38, which promote apoptotic pathway activation [57,58].

## 4. The Key Role of Dietary Supplements in Pregnancy: Multivitamins and Folic Acid

**Figure 2:**
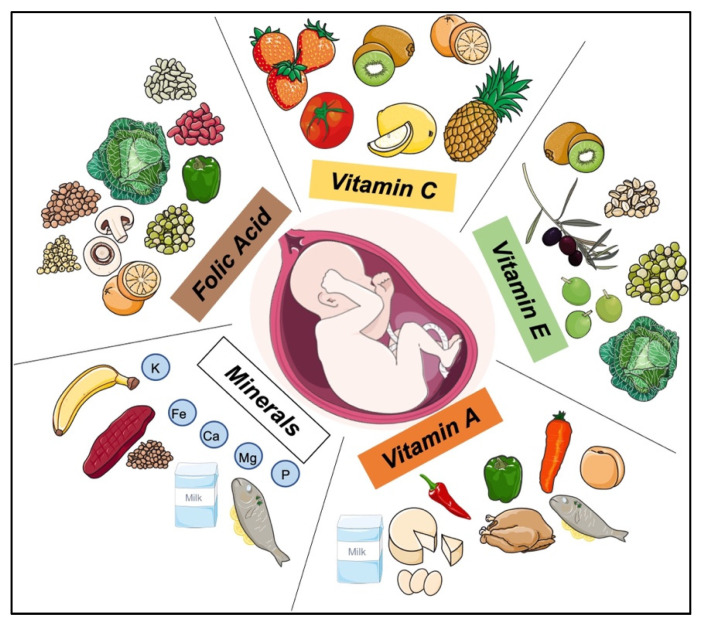
Maternal vitamins and minerals, with related food sources, associated with decreased risk of the development of pediatric cancer. The figure was partly generated using Servier Medical Art, provided by Servier, licensed under a Creative Commons Attribution 3.0 unported license.

Multivitamin dietary supplements contain vitamins and minerals, are administrated with a healthy and balanced diet, but vary in vitamin and mineral contents. Specific multivitamin supplements are tailored to improve athletic performance, reinforce the immune system, facilitate weight control, reduce cardiovascular risk factors, and improve body fluid drainage [108]. During the gestational period of pregnancy, multivitamin supplements are used to offset micronutrient deficiency caused by placental and fetal growth and are important for both maternal and fetal health [63,109]. Multivitamin supplements used in pregnancy contain folic acid, vitamin C, vitamin E, vitamin A, β-carotene, and minerals such as iron, calcium and magnesium (Figure 2) [110], with supplements containing vitamin A and folic acid shown to reduce the incidence of NB by up to 30–40% [19,111].

Interest in vitamin A as an agent to prevent cancer, however, has waned due to its pronounced cytotoxicity [52]. Therefore, retinoids rather than vitamin A have been recommended by the World Health Organization (WHO) as a component of multivitamin supplements to promote fetal and infant health and decrease the risk of NB in pregnant women [63]. The WHO recommends a daily dose of retinol activity equivalents in pregnant and breastfeeding women of 800 μg [63], with vitamin A concentrations of less than 0.70 µmol/L considered to represent subclinical vitamin A deficiency [112].

Folic acid is a synthetic form of the water-soluble B group vitamin folate [113,114], naturally present in several vegetables including legumes, green leafy vegetables, and some fruits [115]. As folate is more expensive and temperature sensitive, folic acid is more widely used in dietary supplements. The active form of folic acid, 5-methyltetrahydrofolate (5-MTHF), is produced in the liver by folic acid reduction [114], is fundamental for embryonic development. 5-MTHF deficiency during pregnancy, promotes premature birth, and increases the risk of neural tube defects (NTDs) [116,117]. In a 1997 study, the consumption of cereal fortified with folic acid was shown to reduce the number of NB diagnoses in Canada, suggesting a potential role for folic acid in preventing NB. This was supported in a subsequent retrospective folic acid fortification study that also reported a reduction in NB cases [118], later confirmed by a meta-analysis of previous data concerning NB incidence following maternal multivitamin ingestion. However, the different combinations of vitamins and minerals in multivitamin supplements has hampered the identification of the actual components that reduce NB incidence, and no studies have been reported that demonstrate a relationship between a specific multivitamin supplement and NB pathogenesis [119].

## 5. Discussion and Conclusions

The majority of NBs are diagnosed in advanced stage, and despite aggressive multimodal therapeutic strategies, continue to exhibit a high frequency of post therapeutic relapse, poor prognosis, and low overall survival, making an effective cure highly challenging [30,33]. Although novel targeted therapies may eventually improve response rates and outcomes in advanced stage NB, novel maternal nutritional supplements containing the NB inhibitory nutraceuticals and vitamins reviewed in this article, provide an important additional strategy to reduce NB development, enhance spontaneous regression, and reduce the early progression of NBs during pregnancy and breast feeding. This potential would add to the important aims of using maternal nutritional supplements to augment maternal, fetal, and childhood health and may also extend to the prevention of additional fetal pathologies, exemplified by folic acid prevention of neural tube defects [117,118]. For these potentials to be realized, problems associated with nutraceutical bioavailability, toxicity, and the route of administration must be addressed and resolved [52,75].

In conclusion, the use of novel maternal supplements containing NB inhibitory nutraceuticals and vitamins not only has the potential to prevent NB development, but also to enhance spontaneous NB regression and reduce early NB progression during fetal growth and early childhood. This novel underestimated approach provides a potentially important additional weapon in the battle to defeat this highly challenging pediatric cancer and, therefore, warrants complete and thorough investigation.

## Figures and Tables

**Table 1:** Overview of the anticancer activities of nutraceuticals in NB models.

Bioactive Compound	Main Source(s)	Molecular Mechanism/Target(s)	Reference
Curcumin	Turmeric	Inhibition of cell growth and apoptosis induction by p53 upregulation	[37]
		Apoptosis induction by PTEN upregulation	[38]
		Apoptosis induction by HSP60 downregulation	[39]
		Inhibition of tumor progression and migration by Atx downregulation	[40]
		Inhibition of tumor cell migration	[8]
		Inhibition of cell migration and invasion by MMP-2 downregulation and TIMP1 activation	[41]
Resveratrol	Grape skin and seeds, wine, peanuts, tea and berries	Apoptosis induction by caspase 3 activation, cell cycle arrest at S-phase by cyclin E upregulation and p21 downregulation	[42]
		Apoptosis induction	[43]
		Apoptosis induction and inhibition of cell proliferation by p53 activation	[44]
		Inhibition of oncogenic signals through prevention of ROS production and TrkAIII activation	[45]
Allicin	Garlic	Apoptosis induction and cell proliferation inhibition by ODC1 downregulation	[46]
S-allylcysteine (SAC)		Apoptosis induction and cell growth inhibition through mitochondrial permeability induction	[47]
Diallyl disulfide (DADS)		Apoptosis induction and cell proliferation inhibition	[48]
		Cytoskeletal damage, G2/M cell cycle arrest and apoptosis induction	[49]
		Anti-apoptotic effect by PGC1α activation	[50]
All-trans retinoic acid (ATRA)	Vitamin A metabolite– Animal products, vegetables and fruits	Differentiation activity	[51]
9-cis-retinoic acid (9-cis-RA)		Reduction of tumor growth in vivo	[52]
		Differentiation effect in amplified NMYC cell lines	[53]
Epigallocatechin-3-gallate (EGCG)	Green tea	Apoptosis induction through caspase and calpain activation	[54]
		Inhibition of tumor relapse and resistance to chemotherapy	[55]
		Enhanced efficacy in combination with 6-OH-11-O-hydroxyphenanthrene	[56]
Bergamot essential oil: BEO, BEO-TF, BEO-TFi	Bergamot (Citrus)	Apoptosis induction by p53 phosphorylation, increased of Bax and decrease of Bcl-2, reduced phosphorylation of p38	[57]
Bergamottin, 5-Geranyloxy-7-methoxycoumarin		Increase apoptosis and ROS levels, increase of BAX, p53, CASP9 and CASP3, decrease of Bcl-2 and Bcl-XL	[58]
Caffeine	Coffee, tea	Apoptotic induction by increasing caspase-3 activity	[59]
Berberine (BRB)	Medicinal plants	Enhanced apoptosis p53-mediated	[60]
		Apoptosis induction in combination with As_2_O_3_	[61]
		Promoting differentiation through increased expression of MAP2, β-III tubulin, NCAM and downregulation of stemness marker as CD133, beta-catenin, notch2, MYCN, and SOX2	[62]

## Data Availability

Citations of relevance to this manuscript were selected from PubMed and Google Scholar data banks, integrating search terms including cancer, tumor, neuroblastoma, nutraceuticals, and all of the individual natural foods, nutraceutical compounds, and vitamins reviewed in this manuscript. Citations also include relevant publications originating from our own research, available upon reasonable request.

## References

[1] Chung C., Boterberg T., Lucas J., Panoff J., Valteau-Couanet D., Hero B., Bagatell R., Hill-Kayser C.E. (2021). Neuroblastoma. Pediatr. Blood Cancer.

[2] Newman E.A., Abdessalam S., Aldrink J.H., Austin M., Heaton T.E., Bruny J., Ehrlich P., Dasgupta R., Baertschiger R.M., Lautz T.B. (2019). Update on Neuroblastoma. J. Pediatr. Surg..

[3] Gatta G., Ferrari A., Stiller C.A., Pastore G., Bisogno G., Trama A., Capocaccia R. (2012). Embryonal Cancers in Europe. Eur. J. Cancer.

[4] Lakhoo K., Sowerbutts H. (2010). Neonatal Tumours. Pediatr. Surg. Int..

[5] Johnsen J.I., Dyberg C., Wickström M. (2019). Neuroblastoma- A Neural Crest Derived Embryonal Malignancy. Front. Mol. Neurosci..

[6] Kholodenko I.V., Kalinovsky D.V., Doronin I.I., Deyev S.M., Kholodenko R.V. (2018). Neuroblastoma Origin and Therapeutic Targets for Immunotherapy. J. Immunol. Res..

[7] Baker D.L., Schmidt M.L., Cohn S.L., Maris J.M., London W.B., Buxton A., Stram D., Castleberry R.P., Shimada H., Sandler A. (2011). Outcome after Reduced Chemotherapy for Intermediate-Risk Neuroblastoma. N. Engl. J. Med..

[8] Zhai K., Brockmüller A., Kubatka P., Shakibaei M., Büsselberg D. (2020). Curcumin’s Beneficial Effects on Neuroblastoma: Mechanisms, Challenges, and Potential Solutions. Biomolecules.

[9] Colon N.C., Chung D.H. (2011). Neuroblastoma. Adv. Pediatr..

[10] Heck J.E., Ritz B., Hung R.J., Hashibe M., Boffetta P. (2009). The Epidemiology of Neuroblastoma: A Review. Paediatr. Perinat. Epidemiol..

[11] Yan P., Qi F., Bian L., Xu Y., Zhou J., Hu J., Ren L., Li M., Tang W. (2020). Comparison of Incidence and Outcomes of Neuroblastoma in Children, Adolescents, and Adults in the United States: A Surveillance, Epidemiology, and End Results (Seer) Program Population Study. Med. Sci. Monit..

[12] Nakagawara A., Li Y., Izumi H., Muramori K., Inada H., Nishi M. (2018). Neuroblastoma. Jpn. J. Clin. Oncol..

[13] Matthay K.K., Maris J.M., Schleiermacher G., Nakagawara A., Mackall C.L., Diller L., Weiss W.A. (2016). Neuroblastoma. Nat. Rev. Dis. Prim..

[14] Mossè Y.P., Laudenslager M., Longo L., Cole K.A., Wood A., Attiyeh E.F., Laquaglia M.J., Sennett R., Lynch J.E., Perri P. (2008). Identification of ALK as a major familial neuroblastoma predisposition gene. Nature..

[15] Parodi S., Merlo D.F., Ranucci A., Miligi L., Benvenuti A., Rondelli R., Magnani C., Haupt R. (2014). Risk of Neuroblastoma, Maternal Characteristics and Perinatal Exposures: The SETIL Study. Cancer Epidemiol..

[16] Heck J.E., Park A.S., Qiu J., Cockburn M., Ritz B. (2013). An Exploratory Study of Ambient Air Toxics Exposure in Pregnancy and the Risk of Neuroblastoma in Offspring. Environ. Res..

[17] Müller-Schulte E., Kurlemann G., Harder A. (2018). Tobacco, Alcohol and Illicit Drugs during Pregnancy and Risk of Neuroblastoma: Systematic Review. Arch. Dis. Child. Fetal Neonatal Ed..

[18] Serres F., Carney S.L. (2006). Nicotine Regulates SH-SY5Y Neuroblastoma Cell Proliferation through the Release of Brain-Derived Neurotrophic Factor. Brain Res..

[19] Daniels J.L., Olshan A.F., Pollock B.H., Shah N.R., Stram D.O. (2002). Breast-Feeding and Neuroblastoma, USA and Canada. Cancer Causes Control.

[20] Simões-Costa M., Bronner M.E. (2013). Insights into Neural Crest Development and Evolution from Genomic Analysis. Genome Res..

[21] Tsubota S., Kadomatsu K. (2018). Origin and Initiation Mechanisms of Neuroblastoma. Cell Tissue Res..

[22] Marshall G.M., Carter D.R., Cheung B.B., Liu T., Mateos M.K., Meyerowitz J.G., Weiss W.A. (2014). The Prenatal Origins of Cancer. Nat. Rev. Cancer.

[23] Castel V., Grau E., Noguera R., Martínez F. (2007). Molecular Biology of Neuroblastoma. Clin. Transl. Oncol..

[24] Lee J.W., Son M.H., Cho H.W., Ma Y.E., Yoo K.H., Sung K.W., Koo H.H. (2018). Clinical Significance of MYCN Amplification in Patients with High-Risk Neuroblastoma. Pediatr. Blood Cancer.

[25] Tacconelli A., Farina A.R., Cappabianca L., Desantis G., Tessitore A., Vetuschi A., Sferra R., Rucci N., Argenti B., Screpanti I. (2004). TrkA Alternative Splicing: A Regulated Tumor-Promoting Switch in Human Neuroblastoma. Cancer Cell.

[26] Farina A.R., Cappabianca L., Ruggeri P., Gneo L., Pellegrini C., Fargnoli M.C., Mackay A.R. (2018). The Oncogenic Neurotrophin Receptor Tropomyosin-Related Kinase Variant, TrkAIII. J. Exp. Clin. Cancer Res..

[27] Jaboin J., Wild J., Hamidi H., Khanna C., Kim C.J., Robey R., Bates S.E., Thiele C.J. (2002). MS-27-275, an Inhibitor of Histone Deacetylase, Has Marked in vitro and in vivo Antitumor Activity against Pediatric Solid Tumors. Cancer Res..

[28] Trigg R.M., Turner S.D. (2018). ALK in Neuroblastoma: Biological and Therapeutic Implications. Cancers.

[29] Swift C.C., Eklund M.J., Kraveka J.M., Alazraki A.L. (2018). Updates in Diagnosis, Management, and Treatment of Neuroblastoma. Radiographics.

[30] Cohn S.L., Pearson A.D.J., London W.B., Monclair T., Ambros P.F., Brodeur G.M., Faldum A., Hero B., Iehara T., Machin D. (2009). The International Neuroblastoma Risk Group (INRG) Classification System: An INRG Task Force Report. J. Clin. Oncol..

[31] Pinto N.R., Applebaum M.A., Volchenboum S.L., Matthay K.K., London W.B., Ambros P.F., Nakagawara A., Berthold F., Schleiermacher G., Park J.R. (2015). Advances in Risk Classification and Treatment Strategies for Neuroblastoma. J. Clin. Oncol..

[32] Mora J. (2022). Autologous Stem-Cell Transplantation for High-Risk Neuroblastoma: Historical and Critical Review. Cancers..

[33] Heinly B.E., Grant C.N. (2022). Cell Adhesion Molecules in Neuroblastoma: Complex Roles, Therapeutic Potential. Front. Oncol..

[34] Treis D., Umapathy G., Fransson S., Guan J., Mendoza-García P., Siaw J.T., Wessman S., Gordon Murkes L., Stenman J., Djos A. (2022). Sustained Response to Entrectinib in an Infant with a Germline ALKAL2 Variant and Refractory Metastatic Neuroblastoma with Chromosomal 2p Gain and Anaplastic Lymphoma Kinase and Tropomyiosin Receptor Kinase Activation. JCO Precis. Oncol..

[35] Kalra E.K. (2003). Nutraceutical—Definition and Introduction. AAPS Pharm. Sci..

[36] Maiuolo J., Gliozzi M., Carresi C., Musolino V., Oppedisano F., Scarano F., Nucera S., Scicchitano M., Bosco F., Macri R. (2021). Nutraceuticals and Cancer: Potential for Natural Polyphenols. Nutrients.

[37] Liontas A., Yeger H. (2004). Curcumin and Resveratrol Induce Apoptosis and Nuclear Translocation and Activation of p53 in Human Neuroblastoma. Anticancer Res..

[38] Picone P., Nuzzo D., Caruana L., Messina E., Scafidi V., Di Carlo M. (2014). Curcumin Induces Apoptosis in Human Neuroblastoma Cells via Inhibition of AKT and Foxo3a Nuclear Translocation. Free Radic. Res..

[39] Bavisotto C.C., Gammazza A.M., Lo Cascio F., Mocciaro E., Vitale A.M., Vergilio G., Pace A., Cappello F., Campanella C., Piccionello A.P. (2020). Curcumin Affects HSP60 Folding Activity and Levels in Neuroblastoma Cells. Int. J. Mol. Sci..

[40] Farina A.R., Cappabianca L., Ruggeri P., Di Ianni N., Ragone M., Merolle S., Sano K., Stracke M.L., Horowitz J.M., Gulino A. (2012). Constitutive Autotaxin Transcription by Nmyc-Amplified and Non-Amplified Neuroblastoma Cells Is Regulated by a Novel AP-1 and SP-Mediated Mechanism and Abrogated by Curcumin. FEBS Lett..

[41] Namkaew J., Jaroonwitchawan T., Rujanapun N., Saelee J., Noisa P. (2018). Combined Effects of Curcumin and Doxorubicin on Cell Death and Cell Migration of SH-SY5Y Human Neuroblastoma Cells. Vitr. Cell. Dev. Biol.-Anim..

[42] Chen Y., Tseng S.H., Lai H.S., Chen W.J. (2004). Resveratrol-Induced Cellular Apoptosis and Cell Cycle Arrest in Neuroblastoma Cells and Antitumor Effects on Neuroblastoma in Mice. Surgery.

[43] Van Ginkel P.R., Sareen D., Subramanian L., Walker Q., Darjatmoko S.R., Lindstrom M.J., Kulkarni A., Albert D.M., Polans A.S. (2007). Resveratrol Inhibits Tumor Growth of Human Neuroblastoma and Mediates Apoptosis by Directly Targeting Mitochondria. Clin. Cancer Res..

[44] Pizarro J.G., Verdaguer E., Ancrenaz V., Junyent F., Sureda F., Pallàs M., Folch J., Camins A. (2011). Resveratrol Inhibits Proliferation and Promotes Apoptosis of Neuroblastoma Cells: Role of Sirtuin 1. Neurochem. Res..

[45] Farina A.R., Cappabianca L., Gneo L., Ruggeri P., Mackay A.R. (2018). TrkAIII Signals Endoplasmic Reticulum Stress to the Mitochondria in Neuroblastoma Cells, Resulting in Glycolytic Metabolic Adaptation. Oncotarget.

[46] Schultz C.R., Gruhlke M.C.H., Slusarenko A.J., Bachmann A.S. (2020). Allicin, a Potent New Ornithine Decarboxylase Inhibitor in Neuroblastoma Cells. J. Nat. Prod..

[47] Kanamori Y., Dalla Via L., Macone A., Canettieri G., Greco A., Toninello A., Agostinelli E. (2020). Aged Garlic Extract and Its Constituent, S-allyl-L-cysteine, Induce the Apoptosis of Neuroblastoma Cancer Cells Due to Mitochondrial Membrane Depolarization. Exp. Ther. Med..

[48] Filomeni G., Aquilano K., Rotilio G., Ciriolo M.R. (2003). Reactive Oxygen Species-Dependent c-Jun NH2-Terminal Kinase/c-Jun Signaling Cascade Mediates Neuroblastoma Cell Death Induced by Diallyl Disulfide. Cancer Res..

[49] Aquilano K., Vigilanza P., Filomeni G., Rotilio G., Ciriolo M.R. (2010). Tau Dephosphorylation and Microfilaments Disruption Are Upstream Events of the Anti-Proliferative Effects of DADS in SH-SY5Y Cells. J. Cell. Mol. Med..

[50] Pagliei B., Aquilano K., Baldelli S., Ciriolo M.R. (2013). Garlic-Derived Diallyl Disulfide Modulates Peroxisome Proliferator Activated Receptor Gamma Co-Activator 1 Alpha in Neuroblastoma Cells. Biochem. Pharmacol..

[51] Preis P.N., Saya H., Nadasdi L., Hochhaus G., Levin V., Sadee W. (1988). Neuronal Cell Differentiation of Human Neuroblastoma Cells by Retinoic Acid plus Herbimycin A. Cancer Res..

[52] Ponthan F., Borgström P., Hassan M., Wassberg E., Redfern C.P.F., Kogner P. (2001). The Vitamin A Analogues: 13-Cis Retinoic Acid, 9-Cis Retinoic Acid, and Ro 13-6307 Inhibit Neuroblastoma Tumour Growth in Vivo. Med. Pediatr. Oncol..

[53] Han G., Chang B., Connor M.J., Sidell N. (1995). Enhanced Potency of 9-Cis versus All-Trans-Retinoic Acid to Induce the Differentiation of Human Neuroblastoma Cells. Differentiation.

[54] Das A., Banik N.L., Ray S.K. (2006). Mechanism of Apoptosis with the Involvement of Calpain and Caspase Cascades in Human Malignant Neuroblastoma SH-SY5Y Cells Exposed to Flavonoids. Int. J. Cancer.

[55] Nishimura N., Hartomo T.B., Van Huyen Pham T., Lee M.J., Yamamoto T., Morikawa S., Hasegawa D., Takeda H., Kawasaki K., Kosaka Y. (2012). Epigallocatechin Gallate Inhibits Sphere Formation of Neuroblastoma BE(2)-C Cells. Environ. Health Prev. Med..

[56] Farabegoli F., Govoni M., Spisni E., Papi A. (2018). Epigallocatechin-3-Gallate and 6-OH-11-O-Hydroxyphenanthrene Limit BE(2)-C Neuroblastoma Cell Growth and Neurosphere Formation in Vitro. Nutrients.

[57] Navarra M., Ferlazzo N., Cirmi S., Trapasso E., Bramanti P., Lombardo G.E., Minciullo P.L., Calapai G., Gangemi S. (2015). Effects of Bergamot Essential Oil and Its Extractive Fractions on SH-SY5Y Human Neuroblastoma Cell Growth. J. Pharm. Pharmacol..

[58] Maugeri A., Lombardo G.E., Musumeci L., Russo C., Gangemi S., Calapai G., Cirmi S., Navarra M. (2021). Bergamottin and 5-Geranyloxy-7-Methoxycoumarin Cooperate in the Cytotoxic Effect of *Citrus bergamia* (Bergamot) Essential Oil in Human Neuroblastoma SH-SY5Y Cell Line. Toxins.

[59] Jang M.H., Shin M.C., Kang I.S., Baik H.H., Cho Y.H., Chu J.P., Kim E.H., Kim C.J. (2002). Caffeine Induces Apoptosis in Human Neuroblastoma Cell Line SK-N-MC. J. Korean Med. Sci..

[60] Choi M.S., Yuk D.Y., Oh J.H., Jung H.Y., Han S.B., Moon D.C., Hong J.T. (2008). Berberine Inhibits Human Neuroblastoma Cell Growth through Induction of p53-Dependent Apoptosis. Anticancer Res..

[61] Kim D.W., Ahan S.H., Kim T.Y. (2007). Enhancement of Arsenic Trioxide (As(2)O(3))- Mediated Apoptosis Using Berberine in Human Neuroblastoma SH-SY5Y Cells. J. Korean Neurosurg. Soc..

[62] Naveen C.R., Gaikwad S., Agrawal-Rajput R. (2016). Berberine Induces Neuronal Differentiation through Inhibition of Cancer Stemness and Epithelial-Mesenchymal Transition in Neuroblastoma Cells. Phytomedicine.

[63] Keats E.C., Haider B.A., Tam E., Bhutta Z.A. (2019). Multiple-Micronutrient Supplementation for Women during Pregnancy. Cochrane Database Syst. Rev..

[64] Pulido-Moran M., Moreno-Fernandez J., Ramirez-Tortosa C., Ramirez-Tortosa M.C. (2016). Curcumin and Health. Molecules.

[65] Kotha R.R., Luthria D.L. (2019). Curcumin: Biological, Pharmaceutical, Nutraceutical, and Analytical Aspects. Molecules.

[66] Nelson K.M., Dahlin J.L., Bisson J., Graham J., Pauli G.F., Walters M.A. (2017). The Essential Medicinal Chemistry of Curcumin. J. Med. Chem..

[67] Prasad S., Gupta S.C., Tyagi A.K., Aggarwal B.B. (2014). Curcumin, a Component of Golden Spice: From Bedside to Bench and Back. Biotechnol. Adv..

[68] Kuttan R., Bhanumathy P., Nirmala K., George M.C. (1985). Potential Anticancer Activity of Turmeric (*Curclima longa*). Cancers Lett..

[69] Srimal R.C., Dhawan B.N. (1973). Pharmacology of Diferuloyl Methane (Curcumin), a Non-Steroidal Anti-Inflammatory Agent. J. Pharm. Pharmacol..

[70] Satoskar R.R., Shah S.J., Shenoy S.G. (1986). Evaluation of Anti-Inflammatory Property of Curcumin (Diferuloyl Methane) in Patients with Postoperative Inflammation. Int. J. Clin. Pharmacol. Ther. Toxicol..

[71] Sharma O.P. (1976). Antioxidant Activity of Curcumin and Related Compounds. Biochem. Pharmacol..

[72] Negi P.S., Jayaprakasha G.K., Rao L.J.M., Sakariah K.K. (1999). Antibacterial Activity of Turmeric Oil: A Byproduct from Curcumin Manufacture. J. Agric. Food Chem..

[73] Maheshwari R.K., Singh A.K., Gaddipati J., Srimal R.C. (2006). Multiple Biological Activities of Curcumin: A Short Review. Life Sci..

[74] Anand P., Kunnumakkara A.B., Newman R.A., Aggarwal B.B. (2007). Bioavailability of Curcumin: Problems and Promises. Mol. Pharm..

[75] Ma Z., Wang N., He H., Tang X. (2019). Pharmaceutical Strategies of Improving Oral Systemic Bioavailability of Curcumin for Clinical Application. J. Control. Release.

[76] Burns J., Yokota T., Ashihara H., Lean M.E.J., Crozier A. (2002). Plant Foods and Herbal Sources of Resveratrol. J. Agric. Food Chem..

[77] Galiniak S., Aebisher D., Bartusik-Aebisher D. (2019). Health Benefits of Resveratrol Administration. Acta Biochim. Pol..

[78] Tian B., Liu J. (2020). Resveratrol: A Review of Plant Sources, Synthesis, Stability, Modification and Food Application. J. Sci. Food Agric..

[79] Shrikanta A., Kumar A., Govindaswamy V. (2015). Resveratrol Content and Antioxidant Properties of Underutilized Fruits. J. Food Sci. Technol..

[80] Gülçin I. (2010). Antioxidant Properties of Resveratrol: A Structure-Activity Insight. Innov. Food Sci. Emerg. Technol..

[81] Baek S.H., Ko J.H., Lee H., Jung J., Kong M., Lee J.W., Lee J., Chinnathambi A., Zayed M., Alharbi S.A. (2016). Resveratrol Inhibits STAT3 Signaling Pathway through the Induction of SOCS-1: Role in Apoptosis Induction and Radiosensitization in Head and Neck Tumor Cells. Phytomedicine.

[82] Ren B., Kwah M.X.Y., Liu C., Ma Z., Shanmugam M.K., Ding L., Xiang X., Ho P.C.L., Wang L., Ong P.S. (2021). Resveratrol for Cancer Therapy: Challenges and Future Perspectives. Cancer Lett..

[83] Soto B.L., Hank J.A., Van De Voort T.J., Subramanian L., Polans A.S., Rakhmilevich A.L., Yang R.K., Seo S., Kim K., Reisfeld R.A. (2011). The Anti-Tumor Effect of Resveratrol Alone or in Combination with Immunotherapy in a Neuroblastoma Model. Cancer Immunol. Immunother..

[84] Zhang Y., Liu X., Ruan J., Zhuang X., Zhang X., Li Z. (2020). Phytochemicals of Garlic: Promising Candidates for Cancer Therapy. Biomed. Pharmacother..

[85] Petrovska B., Cekovska S. (2010). Extracts from the History and Medical Properties of Garlic. Pharmacogn. Rev..

[86] Chen J., Huang G. (2019). Antioxidant Activities of Garlic Polysaccharide and Its Phosphorylated Derivative. Int. J. Biol. Macromol..

[87] Jonkers D., Van Den Broek E., Van Dooren I., Thijs C., Dorant E., Hageman G., Stobberingh E. (1999). Antibacterial Effect of Garlic and Omeprazole on Helicobacter Pylori. J. Antimicrob. Chemother..

[88] Ried K., Frank O.R., Stocks N.P., Fakler P., Sullivan T. (2008). Effect of Garlic on Blood Pressure: A Systematic Review and Meta-Analysis. BMC Cardiovasc. Disord..

[89] Patiño-Morales C.C., Jaime-Cruz R., Sánchez-Gómez C., Corona J.C., Hernández-Cruz E.Y., Kalinova-Jelezova I., Pedraza-Chaverri J., Maldonado P.D., Silva-Islas C.A., Salazar-García M. (2022). Antitumor Effects of Natural Compounds Derived from *Allium sativum* on Neuroblastoma: An Overview. Antioxidants.

[90] Majewski M. (2014). *Allium sativum*: Facts and Myths Regarding Human Health. Rocz. Państwowego Zakładu Hig..

[91] Iciek M., Kwiecień I., Włodek L. (2009). Biological Properties of Garlic and Garlic-Derived Organosulfur Compounds. Environ. Mol. Mutagen..

[92] Gao X.Y., Geng X.J., Zhai W.L., Zhang X.W., Wei Y., Hou G.J. (2015). Effect of Combined Treatment with Cyclophosphamidum and Allicin on Neuroblastoma-Bearing Mice. Asian Pac. J. Trop. Med..

[93] Garry P.J. (1981). Vitamin A. Clin. Lab. Med..

[94] Siddikuzzaman, Guruvayoorappan C., Berlin Grace V.M. (2011). All Trans Retinoic Acid and Cancer. Immunopharmacol. Immunotoxicol..

[95] Koprivica M., Bjelanovic J. (2021). The Importance of Vitamin A in the Nutrition. Med. Cas..

[96] Carazo A., Macáková K., Matoušová K., Krčmová L.K., Protti M., Mladěnka P. (2021). Vitamin a Update: Forms, Sources, Kinetics, Detection, Function, Deficiency, Therapeutic Use and Toxicity. Nutrients.

[97] Chelstowska S., Widjaja-Adhi M.A.K., Silvaroli J.A., Golczak M. (2016). Molecular Basis for Vitamin A Uptake and Storage in Vertebrates. Nutrients.

[98] Lynch S., Pfeiffer C.M., Georgieff M.K., Brittenham G., Fairweather-Tait S., Hurrell R.F., McArdle H.J., Raiten D.J. (2018). Biomarkers of Nutrition for Development (BOND)-Iron Review. J. Nutr..

[99] McLaren D.S., Kraemer K. (2012). Vitamin A in Health. World Rev. Nutr. Diet..

[100] Duester G. (2008). Retinoic Acid Synthesis and Signaling during Early Organogenesis. Cell.

[101] Reynolds C.P., Matthay K.K., Villablanca J.G., Maurer B.J. (2003). Retinoid Therapy of High-Risk Neuroblastoma. Cancer Lett..

[102] Reynolds C.P. (2000). Differentiating Agents in Pediatric Malignancies: Retinoids in Neuroblastoma. Curr. Oncol. Rep..

[103] Alvarez S., Germain P., Alvarez R., Rodríguez-Barrios F., Gronemeyer H., de Lera A.R. (2007). Structure, Function and Modulation of Retinoic Acid Receptor Beta, a Tumor Suppressor. Int. J. Biochem. Cell Biol..

[104] Bayeva N., Coll E., Piskareva O. (2021). Differentiating Neuroblastoma: A Systematic Review of the Retinoic Acid, Its Derivatives, and Synergistic Interactions. J. Pers. Med..

[105] Hossain M.M., Banik N.L., Ray S.K. (2012). Survivin Knockdown Increased Anti-Cancer Effects of (−)-Epigallocatechin-3-Gallate in Human Malignant Neuroblastoma SK-N- BE2 and SH-SY5Y Cells. Exp. Cell Res..

[106] Yang C.S., Wang X., Lu G., Picinich S.C. (2009). Cancer Prevention by Tea: Animal Studies, Molecular Mechanisms and Human Relevance. Nat. Rev. Cancer.

[107] Calvani M., Subbiani A., Bruno G., Favre C., Gil G. (2020). Beta-Blockers and Berberine: A Possible Dual Approach to Contrast Neuroblastoma Growth and Progression. Oxidative Med. Cell. Longev..

[108] National Institutes of Health (NIH) Multivitamin/Mineral Supplements—Health Professional Fact Sheet. https://ods.od.nih.gov/factsheets/MVMS-HealthProfessional/.

[109] Yakoob M.Y., Khan Y.P., Bhutta Z.A. (2010). Maternal Mineral and Vitamin Supplementation in Pregnancy. Expert Rev. Obstet. Gynecol..

[110] Rios P., Bailey H.D., Orsi L., Lacour B., Valteau-Couanet D., Levy D., Corradini N., Leverger G., Defachelles A.S., Gambart M. (2016). Risk of Neuroblastoma, Birth-Related Characteristics, Congenital Malformations and Perinatal Exposures: A Pooled Analysis of the ESCALE and ESTELLE French Studies (SFCE). Int. J. Cancer.

[111] Milne E., Greenop K.R., Bower C., Miller M., Van Bockxmeer F.M., Scott R.J., De Klerk N.H., Ashton L.J., Gottardo N.G., Armstrong B.K. (2012). Maternal Use of Folic Acid and Other Supplements and Risk of Childhood Brain Tumors. Cancer Epidemiol. Biomarkers Prev..

[112] Black R.E., Victora C.G., Walker S.P., Bhutta Z.A., Christian P., De Onis M., Ezzati M., Grantham-Mcgregor S., Katz J., Martorell R. (2013). Maternal and Child Undernutrition and Overweight in Low-Income and Middle-Income Countries. Lancet.

[113] Crider K.S., Bailey L.B., Berry R.J. (2011). Folic Acid Food Fortification-Its History, Effect, Concerns, and Future Directions. Nutrients.

[114] Ferrazzi E., Tiso G., Di Martino D. (2020). Folic Acid versus 5- Methyl Tetrahydrofolate Supplementation in Pregnancy. Eur. J. Obstet. Gynecol. Reprod. Biol..

[115] Allen L.H. (2008). Causes of Vitamin B_12_ and Folate Deficiency. Food Nutr Bull..

[116] Valentin M., Coste Mazeau P., Zerah M., Ceccaldi P.F., Benachi A., Luton D. (2018). Acid Folic and Pregnancy: A Mandatory Supplementation. Ann. Endocrinol..

[117] Iyer R., Tomar S.K. (2009). Folate: A Functional Food Constituent. J. Food Sci..

[118] French A.E., Grant R., Weitzman S., Ray J.G., Vermeulen M.J., Sung L., Greenberg M., Koren G. (2003). Folic Acid Food Fortification is Associated with a Decline in Neuroblastoma. Clin. Pharmacol. Ther..

[119] Goh Y.I., Bollano E., Einarson T.R., Koren G. (2007). Prenatal Multivitamin Supplementation and Rates of Pediatric Cancers: A Meta-analysis. Clin. Pharmacol. Ther..

